# Traumatic tattoos mimicking pigmented skin tumors on videodermoscopy: A case series

**DOI:** 10.1111/jocd.16502

**Published:** 2024-08-04

**Authors:** Patrycja Rogowska, Michał Sobjanek, Aneta Szczerkowska‐Dobosz, Roman J. Nowicki, Martyna Sławińska

**Affiliations:** ^1^ Department of Dermatology, Venereology and Allergology, Faculty of Medicine Medical University of Gdańsk Gdańsk Poland

To the Editor,

Traumatic tattooing is defined as the accidental implantation of dark pigments, such as metal or graphite, into the dermis, which usually occurs after road accidents, gunshots, or fireworks explosions. People working in the building and mining industries are more prone to develop traumatic tattoos.^1^ We report four male patients with posttraumatic tattoos, which emerged during videodermoscopic examination as unidentified pigmented skin lesions. While nodular melanoma, blue nevus, and pigmented basal cell carcinoma were initially considered in all cases, further inquiry revealed that the majority of patients reported the lesion appeared subsequent to trauma. In one patient, pigmented hidrocystoma was also suspected as the lesion was localized within the lower eyelid, which is a characteristic location for this tumor. Nevertheless, an uncharacteristic dermoscopic image and the lack of typical dermoscopic features, such as arborizing vessels in basal cell carcinoma, led us to consider the different nature of these lesions.

The first patient, a 49‐year‐old man with multiple melanocytic lesions and a positive history of pigmented basal cell carcinoma presented with a blue nodule on his left forearm. Videodermoscopy showed a combination of structureless blue, white, and brown areas and polymorphic vessels (Figure 1A,B). The patient admitted that the lesion occurred after the accidental implantation of a graphite pencil tip many years before.

**FIGURE 1:**
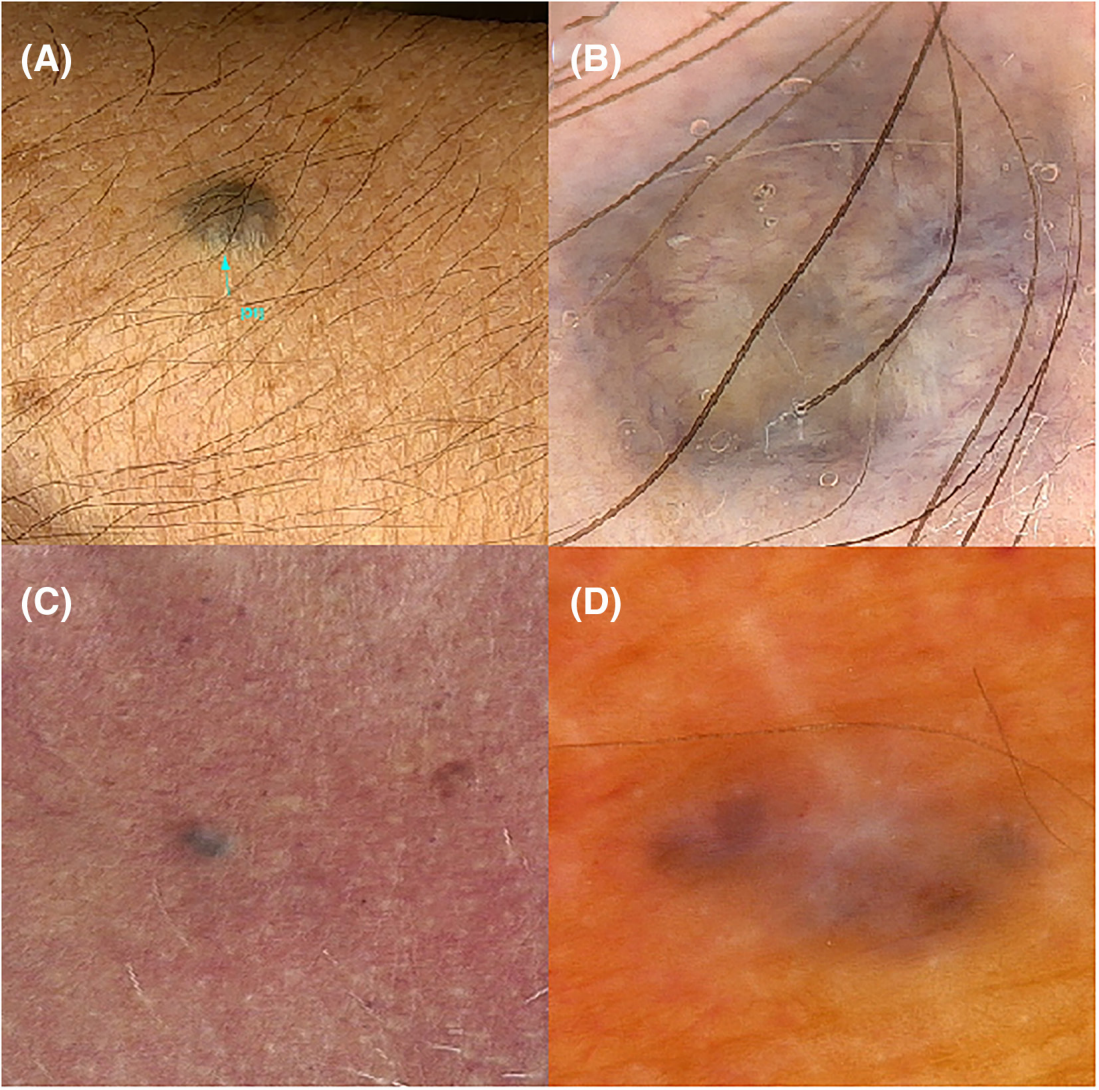
(A) Patient 1. Clinical presentation of a blue nodule on the left forearm (graphite pencil tip); (B) Videodermoscopy showed a combination of structureless blue, white and brown areas and polymorphic vessels (FotoFinder, Medicam 800 HD, ×20 magnification). (C) Patient 2. Clinical presentation of a blue nodule on the chest (metal filling). (D) Videodermoscopy showed white, blue, and brown structureless areas (FotoFinder, Medicam 800 HD, ×20 magnification).

The second patient, a 72‐year‐old patient with a previous history of basal cell carcinoma, presented with a blue nodule on the chest. White, blue, and brown structureless areas were observed on videodermoscopy (Figure 1C,D). The lesion appeared due to an accident while the patient had been grinding metal about 15 years before.

The third patient, an 89‐year‐old man with a history of multiple actinic keratosis lesions and basal cell carcinoma on the face and scalp presented with a blue papule localized within the lower eyelid, with a blue‐brown structureless pattern noticed on videodermoscopic examination (Figure 2A,B). According to the patient, the papule was present for at least 20 years and had been caused by a metal filling.

**FIGURE 2:**
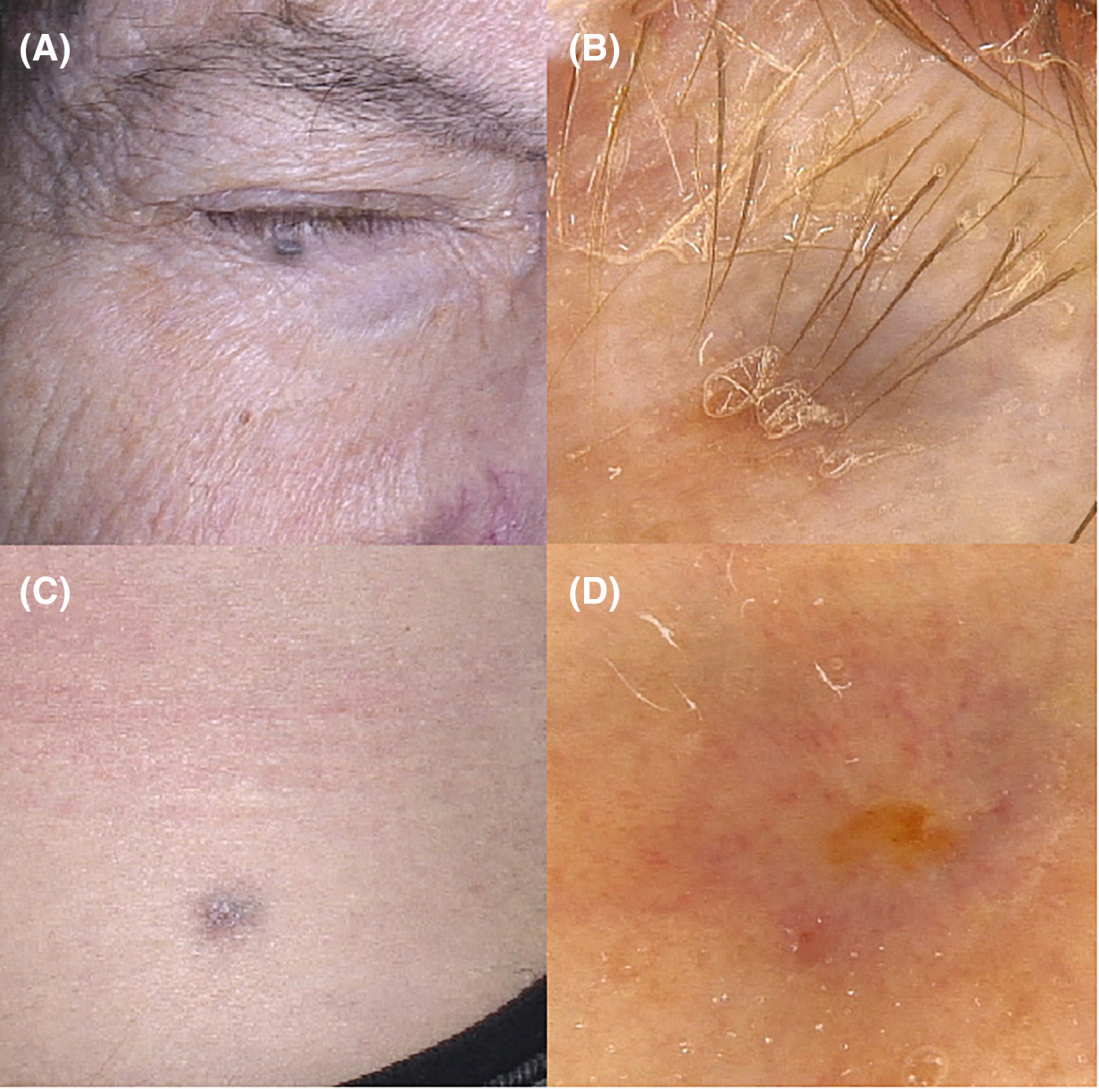
(A) Patient 3. Clinical presentation of a blue papule localized within the right lower eyelid (metal filling). (B) Videodermoscopy showed a blue‐brown structureless pattern (FotoFinder, Medicam 800 HD, ×20 magnification). (C) Patient 4. Clinical presentation of a gray‐pinkish nodule on the inguinal area (gravel). (D) Videodermoscopy showed a central erosion surrounded by multiple linear, globular, and dotted vessels over gray‐brownish background (FotoFinder, Medicam 800 HD, ×20 magnification).

The last patient, a 25‐year‐old motorcycle speedway rider, showed a gray‐pinkish nodule on the inguinal area present for about 2 years. A central erosion surrounded by multiple linear, globular, and dotted vessels over gray‐brownish background was observed on videodermoscopy (Figure 2C,D). A biopsy was taken as the patient was not certain whether the lesion occurred as a consequence of a previous motorbike accident. The diagnosis of a traumatic tattoo was confirmed in the histopathological examination, which showed histiocytic infiltration around the pigment deposits.

There is no specific dermoscopic pattern for traumatic tattoos. Usually, structureless areas can be observed and their color depends on the depth on which ink is located in the skin (similarly to the perception of dermal melanin).^2^ Due to Tyndall's effect, particles located in deep dermis may appear bluish in color.^3^ As the history of trauma is not always apparent, the differentiation between accidental tattoos and pigmented skin tumors can be challenging. Traumatic tattoos may be very similar to e.g. blue nevus, melanoma, or pigmented basal cell carcinoma.^4^ Amalgam tattoos found on the oral mucosa after a dental filling procedure can mimic mucosal melanoma.^5^ We emphasize the importance of medical history containing inquiry about the patient's past accidents, which in some patients can prevent unnecessary invasive diagnostic procedures. Nevertheless, in case of doubts, a biopsy is inevitable to confirm the diagnosis.

## AUTHOR CONTRIBUTIONS

Patrycja Rogowska wrote the manuscript. Patrycja Rogowska, Aneta Szczerkowska‐Dobosz, Michał Sobjanek, Martyna Sławińska, Roman J. Nowicki provided data, conducted the patient interviews and examinations. All authors reviewed the final manuscript.

## CONFLICT OF INTEREST STATEMENT

The authors declare no conflicts of interest with regard to this manuscript.

## ETHICS STATEMENT

The paper accurately reflects the authors’ original research and analysis in a truthful and comprehensive manner.

## CONSENT

Informed consent was obtained from all of the patients presented in the article.

## Data Availability

The data that support the findings of this study are available from the corresponding author upon reasonable request.
